# The Status of Biofilms in Penile Implants

**DOI:** 10.3390/microorganisms5020019

**Published:** 2017-04-18

**Authors:** Matthew Faller, Tobias Kohler

**Affiliations:** Southern Illinois University School of Medicine, Springfield, IL 62702, USA; tkohler@siumed.edu

**Keywords:** prosthetic surgery, penile prosthesis, biofilm, infection

## Abstract

Erectile dysfunction is prevalent among men and will continue to become more so with the aging population. Of the available treatment options, implantable prosthetic devices are typically thought of as a third line treatment even though they have the highest satisfaction rate and continually improving success rates. Infection and mechanical failure are the most common reasons for implant revision in the past. Since the development of more reliable devices, bacterial biofilms are coming to the forefront of discussion as causes of required revision. Biofilms are problematic as they are ubiquitous and exceedingly difficult to prevent or treat.

## 1. Introduction

Bacterial infection has been a longstanding foe of the surgeon and the profession has made great strides to combat this over the centuries. Biofilms are a particular problem for the prosthetic surgeon, but are by no means limited to implantable devices. Biofilms are found on oil and water piping, aquatic structures, teeth, and other indwelling devices such as IV’s and urinary catheters [1]. It is estimated only 0.1% of all the bacterial biomass exists in the planktonic form, which is the classic culprit of infection [2]. With the overwhelming majority of bacteria living in organized colonies and most medically relevant bacteria able to do so [3], it is no surprise they would come to light eventually. As such, it wasn’t until 1977 that biofilm was reported as the cause of a chronic infection [4].

In the field of Urology biofilms cause complications with simple devices such as Foley Catheters [5] as well as the more advanced three piece inflatable penile prosthetic.

Erectile dysfunction (ED) is defined as difficulty achieving or maintaining an erection [6]. It can be the result of pathology in several organ systems including vascular, neurogenic, psychological, and iatrogenic [7]. ED is a growing problem in the United States at least partly due to rising numbers of prostate cancer survivors [8] and also because of the aging population. Treatment usually progresses from oral medications, to directly injectable medications, and the final, permanent option of an implantable penile prosthetic. Penile implants have evolved from the original subcutaneous acrylic implant in 1949 [9], the first three-piece inflatable penile prosthesis (IPP) in 1973 [10], to the modern IPPs used today.

Archaic and uninformed notions that IPPs are terrible devices with risks that far outweigh the benefits [11] are dispelled with current data. IPPs have a positive impact on patient quality of life and complication rates well below acceptable ranges with 20,000 new implants placed every year [12]. In one study by Carson CC, after 3 years 92% of patients had a reliable device and after 5 years 86% of devices were reliable, 79% of patients used it twice monthly, and 88% would recommend it for a friend [13]. A Korean study reported 5, 10 and 15 year implant survival rates of 89.1%, 71.4% and 60.5% with similar results previously published and patient satisfaction rates of >80% [14]. Another study directly compared satisfaction of IPPs compared to oral and injectable medications revealing significantly higher rates for IPPs [15]. Nevertheless, infected IPPs cause patient harm and healthcare dollars. Treating an infected IPP costs up to 6 times the amount of the original procedure [16].

## 2. What Is Biofilm?

Bacteria are classically depicted and studied in their planktonic form. In this state, the bacteria rely on several factors for protection such as a plasma membrane, extracellular matrix, enzymes to inactivate offending agents, and Multi-Drug Resistant efflux pumps. Another pivotal fact about these organisms is that they are easily accessible for study, which is why all current antibiotics were designed for planktonic bacteria [3]. Biofilm colonies are not so easily studied as they are difficult to culture by standard methods and therefore treatment is lagging behind. The definition of biofilm is changing with new data that arises [3], but it can classically be defined as “a structured consortium of bacteria encased in a self-producing matrix [17].” Biofilm is almost always associated with a surface for attachment, but some observations of their formation without a surface for attachment have been made [3]. The general structure of the biofilm has three layers [18]. The linking layer abutting the adherent surface, a compact layer of bacteria, and free-floating bacteria. The biofilm could be viewed both as single cells or a greater, living entity.

A discussion on biofilm can be distilled down to a four step process to enhance understanding; attachment, aggregation and accumulation, maturation, and detachment [19]. Attachment is arguably the most important step in biofilm formation and its process is multifactorial. Forces involved include foundational forces; such as Van der Waals, electrostatic, and hydrophobicity, as well as biological; proteins called autolysins and fimbriae, that help establish interactions [17,18]. Among the most important interactions is hydrophobicity [20]. Hydrophilic coatings create large hydration barriers, which have an exclusion volume effect preventing bacteria from adhering to the surface [17]. The second step, aggregation and accumulation, involves growth of the bacteria leading to the development of layers of cells on the surface. The bacteria are closely associated via intercellular adhesions which induce changes in gene expression, a characteristic known as quorum sensing, which help regulate the lifecycle of the biofilm colony [21]. During maturation the “film” of the biofilm, often referred to as Extracellular Polymeric Substance (EPS), is developed. The EPS is composed of a complex matrix of carbohydrates, proteins, lipids, and DNA [17] (eDNA). DNA has been of particular interest lately with the full details beyond the scope of this article. Sources may differ slightly on exact percentages, but the majority of a mature biofilm colony is composed of EPS with only 15% being biomass [22]. These materials form a structure that has channels for bulk fluids to flow through and carry nutrients and chemical signals much like hormones in the human body. As the colony grows to a critical density, quorum sensing again takes place-causing expression of cleavage enzymes, thus releasing bacteria from the colony [17] resulting in the last step, dispersion. These bacteria are now free to enter the bloodstream and/or seed another location and begin the cycle anew. There is much yet to be discovered and understood about the complex signaling and gene regulation processes that occur in a biofilm.

## 3. Why Biofilm Is Problematic

Biofilms are problematic because they are difficult to prevent and resistant to traditional treatment methods. The heartiness of the biofilm colony could be an evolutionary development secondary to selection pressures from amoebae and bactericidal compounds from competing bacteria [3]. The extracellular material creates a thick barrier that impedes the penetration of antibiotics to the biomass layer [23]. The antibiotic that does reach the bacteria may also be less effective due to their reduced metabolism. Biofilms are slow growing to the point of dormancy in the center of the mass [3]. These dormant cells are sometimes referred to as persister cells because of their ability to remain viable after antibiotic treatment [24]. Notably the etiology of their antibiotic “resistance” is a reduced metabolism and not a genetic mutation, such as in Methicillin Resistant *Staph Aureus*. It has been well established that slower growth correlates to reduced antibiotic efficacy. Another hurdle for antibiotic treatment of an infected prosthetic is the fibrous capsule that forms around the implant. This is a physiological process aimed at preventing the spread of potentially harmful materials. This is well demonstrated in infections caused by organisms such as *Histoplasma capsulatum* and *Mycobacterium tuberculosis*. The unfortunate result in an intentionally placed object has reduced blood flow into the capsule, which therefore causes reduced antibiotic delivery [25]. The summative result of these characteristics is an infection capable of surviving antibiotic concentrations up to 1000 times the concentrations of planktonic bacteria.

Neutrophils are the body’s first responders to local infection. They migrate via chemotactic signals, which probably are lacking in a biofilm [17]. Just as antibiotics have difficulty penetrating the film, these signals may minimally diffuse out of the film. Paired with the physical barrier of the film, neutrophils are unable to reach the bacteria deep within the colony. While biofilm infections are resistant to the human immune system, they are not completely inert. The infection does create an inflammatory response, which unfortunately significantly damages the surrounding tissues [17].

The biofilm colony is an ideal setup for antibiotic resistance to develop for two reasons. When exposed to antibiotics at sub-therapeutic concentrations, a selection pressure has been established and bacteria have the potential to evolve into a resistant strain. It has already been established that biofilms may require exponentially higher concentrations that are likely not reached through conventional methods and would potentially have harmful side effects to the host. The other reason is the close proximity of organisms facilitates horizontal gene transfer coding for antibiotic resistance [3].

## 4. The Role of Biofilm in Urological Prosthetics

Biofilms are nearly ubiquitous among IPPs and it is commonly accepted that inoculation occurs at the time of surgery. In a study of IPPs removed for mechanical, non-infectious, reasons scanning laser microscopy identified biofilms on 8/10 devices [26]. Of the devices that do become infected, the presentation can be either clinical or subclinical [18]. Clinically apparent infections are easily identified as the presentation involves fever, erythema and induration over the device, and possibly frank pus. Subclinical infections have a more indolent course with the only symptom often being chronic pain associated with the device. Subclinical infection due to *S. epidermidis* was the most common presentation before the current era of coated implants and improved techniques, but now clinical infections are more prevalent caused by more virulent bacteria.

In 2001 American Medical Systems (AMS) developed the AMS 700 three-piece IPP with InhibiZone coating. This device is impregnated with the antibiotics minocycline and rifampin and releases them mostly over the next 3 days and tapers for up to 3 weeks [8]. In 2002 Coloplast Corporation released the Titan three-piece IPP with its hydrophilic polyvinylpyrrolidone (PVP) coating that absorbs the aqueous antibiotic bath the device is placed in at the time of surgery. When compared to several other antibiotic combinations, trimethoprim-sulfamethoxazole had the greatest zone of inhibition [27].

The advent of IPPs coated with antibiotics have brought with it a decrease in infection rate by about 50% [28]. Wilson, Costerton, and Salem performed an experiment that showed the rifampin/minocycline combination had minimal to no effect on more virulent bugs such as Pseudomonas [8].

## 5. Current Infection Rates

The initial infection rate of IPPs 30 years ago was 3–5% for primary surgeries and 10% for revisions [8]. With the latest devices and techniques, those rates have effectively halved [29]. Mandava et al. reported primary implant infection rates with non-coated at 2.32% and coated IPPs at 0.89% [30]. Another study reported coated primary implant infection rates of 0 of 223 in non-diabetic patients and 1% for diabetic patients. Implants with non-coated devices were abandoned due to infection rates matching the literature. In the same study, revision procedures without washout had an infection rate of 10% as opposed to those with washout showed 2.45% [31]. In larger studies of primary implants, when the “no touch” method was added to the coated implant, infection rates were reduced from 2% to 0.7% [32] and 0.46% [33]. Eid reported no significant difference in results when comparing the modern AMS coated implants to the Coloplast counterparts [33]. Interestingly, the Coloplast implants were only dipped in saline to activate the hydrophilic coating and the washouts performed were also only saline. In agreement with the above, Eid also noted infected non-coated implants presented subclinically and coated implants presented clinically.

## 6. Current Practices to Reduce Infection

Several practices exists that can help a surgeon reduce infection [29,34]. Aside from standard sterile field precautions, a shorter time in the operating room and minimal foot traffic through the theater can decrease risk. Thus seasoned IPP implanters typically have better outcomes [35]. Trimming the operative area with electric clippers in the operating room (OR) versus the night before or using a razor blade is the preferred method for hair removal. This minimizes the potential for micro-abrasions. As far washing the area, the best results are obtained with an antiseptic wash at home in addition to the preparation in the OR [36]. Treating nares colonized with Staphylococcus before surgery was also shown to reduce infection [37]. Preoperative antibiotics should be given one hour before surgery. Oral and IV medication has been shown to have equivalent penetration into the corpora cavernosa so preference may be based on institution [38]. Although a rare cause of infection, clearing remote infections before undergoing surgery can prevent seeding of the prosthesis by transient bacteremia [39]. Also a shorter hospital stay has less risk for nosocomial infection independent of the cause of admission.

Other techniques exist that are more specific to the Urologic prosthetic surgeon. The no touch method described by Eid [33] minimizes skin contact with the field and implant to reduce exposure to normal flora such as *S. epidermidis*. Immediately prior to placement of the device into the body, the cavity should be vigorously washed with antibiotic solutions to both flush out and/or kill any present bacteria while in the planktonic stage. This step is critical and has been shown to lower infection rates significantly [31]. The washout solution has been studied to determine the ideal method. Hydrogen peroxide may cause damage to the surrounding tissues which would delay overall wound healing [25]. Abouassaly et al. studied washouts with one solution versus several and revealed equal results suggesting the mechanical debridement may be the therapeutic agent [40]. Eid also achieved low infection rates with saline washes [33]. In a study of IPP revisions, Henry et al. reported 43% positive tissue cultures before washout and 25% positive after washout [25].

Salvage of an infected IPP can be a two-stage or one-stage process. The one-stage salvage involves removal of the device, washout, changing of gloves, and then replacing a new device immediately. The major benefits of the one-stage salvage being maintenance of the IPP and one less surgery.

The two-stage salvage involves removal of the infected device, placing a malleable prosthesis at that time, giving a course of antibiotics, then several months later exchanging it for another IPP. This has been shown to be an effective method in isolated scrotal pump infections [41]. Removing a penile prosthetic without an immediate replacement for any reason is complicated by a shortened penis and a more difficult second implant surgery due to scarring of the corporal bodies.

## 7. Future of Biofilm Treatment

Biofilm prevention or treatment has a wide array of novel possibilities including surface modification, antibiotics, EPS degrading enzymes, iron chelation, regulatory pathway interference, antibodies against attachment moieties, ultrasound, bacteriophages, and nanotechnology [3,17,42]. Most of these methods are still under study in vitro and have yet to be taken to clinical trial. The depth of all of these methods is beyond the scope of this article; so a few are briefly mentioned.

Preventing bacterial attachment would be the ideal solution for biofilms. This could be achieved through surface modification, impregnated antibiotics, or a combination [17]. Roughness, free energy, and composition are all qualities of a material that determine ease of bacterial adhesion [42]. Coloplast’s PVP coating is an attempt at this. Heparin is also hydrophilic and additionally inhibits bacterial interactions with fibronectin, which has been documented as a means of attachment [17]. Prevention of attachment would mean the host immune response and traditional antibiotics should be sufficient to clear present bacteria. This would only be feasible in a prophylactic manner because biofilm presence has been documented only 16 h after inoculation [5]. One difficulty to overcome with this method is the concept of surface conditioning [3]. An implant becomes quickly coated with host glycoproteins such as fibronectin that helps facilitate bacterial attachment and may mask any coating placed on the implant.

Ultrasound has been used to break up and release bacteria from biofilms to aid with culturing of biofilm [3]. This may theoretically be useful in the treatment of biofilm in situ to disrupt the colony and expose it to antibiotics. In the field of Urology similar technology is used in Extracorporeal Shock Wave Lithotripsy for management of nephrolithiasis, which may allow for easy transition to the new application. Some concerns with this idea are local tissue damage and potential for bacterial seeding elsewhere from released bacteria. Therefore it would be necessary to administer antibiotics in conjunction.

Some other current methods under study are the enzymes Dispersin B and DNase I, which break down the EPS of biofilm and potentially expose the bacteria to antibiotics or host immune cells [17]. Inhaled recombinant DNase has already showed to decrease viscosity of secretions in patients with cystic fibrosis [3]. Iron is a known necessary mineral for bacterial growth, which begs iron chelation as a possibility to prevent mature biofilms [3]. Unfortunately some bacteria such as Pseudomonas produce siderophores, which sequester iron. Given the propensity for gene transfer, this could become a mechanism for resistance. Chemicals like arginine or nitrates could make current antibiotics more effective by accelerating the metabolism of these colonies.

## 8. Future Studies in Urology

The PROPPER study is the first large, multicenter, prospective study of IPP treatment with the most up to date results to come as the study matures [43]. This study will report findings such as: reasons for surgery, surgical techniques used, surgical outcomes, follow up data, impact on patient quality of life, IPP durability, and complication rates. Current results report radical prostatectomy is the most common etiology of surgery.

## 9. Conclusions

Inflatable penile implants remain a staple in treatment of erectile dysfunction as the definitive and permanent treatment method. Increasing numbers of these devices will be implanted in the upcoming years therefore steps must be taken to ensure their safety and efficacy. Currently, biofilm related infections are the biggest threat to patients and surgeons alike. New developments in techniques and device coatings have made significant improvements in the last 15 years. However infection remains a problem that will likely require a novel solution to fix.
